# Die Landesparlamente im Zeichen der Emergency Politics in der Corona-Krise

**DOI:** 10.1007/s41358-021-00310-2

**Published:** 2022-01-12

**Authors:** Benjamin Höhne

**Affiliations:** 1 Institut für Parlamentarismusforschung (IParl), Mauerstr. 83/84, 10117 Berlin, Deutschland; 2 Arbeitsstelle „Politische Soziologie der Bundesrepublik Deutschland“, Freie Universität zu Berlin, Otto-Suhr-Institut, Ihnestraße 21, 14195 Berlin, Deutschland

**Keywords:** Landesparlamente, Emergency Politics, Corona-Pandemie, Bund-Länder-Konferenz, Parlamentsfunktionen, State parliaments, Emergency politics, Corona-Pandemic, Bund-Länder-Konferenz, Parliamentary functions

## Abstract

Auch wenn Deutschlands Performanz bei den Infektions- und Todeszahlen sowie den wirtschaftlichen und sozialen Folgen in Verbindung mit COVID-19 im EU27-Vergleich als zufriedenstellend bewertet würde, stellt sich die Frage nach der Input-Legitimität durch demokratische Verfahren. Sie wird ausgehend vom Konzept der Emergency Politics von Jonathan White am Beispiel Deutschlands mit seinem Notstandsregime der Bund-Länder-Konferenz und dessen Effekten auf die 16 Landesparlamente aufgeworfen. Anschließend daran wird argumentiert, dass die während der Pandemiewellen in den Jahren 2020 und 2021 vorherrschende Entscheidungsfindung und Beschlussfassung innerhalb der föderalen Staatsordnung einen Rahmen bildete, der den Parlamentsfunktionen weitgehend den Boden entzogen hat. Zentral für die Analyse ist die Darlegung der von der Pandemie evozierten Veränderung des Mehrebenen-Regierens durch die Bund-Länder-Konferenzen. Auswirkungen dieser von der Exekutive dominierten Krisenbewältigungspolitik wird für die auf der Landesebene wichtigsten Parlamentsfunktionen, die Kontrolle durch Mitregieren, Oppositionskontrolle, Gesetzgebung sowie das Herstellen von Öffentlichkeit, diskutiert. Empirische Grundlagen bilden Presseberichte, nichtöffentliche Beschlussvorlagen und Beschlüsse der Bund-Länder-Konferenz sowie Bevölkerungsbefragungen. Der Aufsatz schließt mit der Empfehlung, zukünftige Notstandspolitik institutionell besser vorzubereiten und dabei einen Platz für den Landesparlamentarismus vorzusehen.

## Abstract (en)

Although Germany’s performance in terms of the number of infections and fatalities as well as the economic and social consequences associated with COVID-19 can be considered as satisfactory in an EU27 comparison, it raises the question of input legitimacy through democratic procedures. It draws on the basis of Jonathan White’s concept of emergency politics, using the example of Germany with its emergency regime of the *Bund-Länder-Konferenz* and its impact on the 16 state parliaments. It is then argued that the decision-making during the pandemic waves in 2020 and 2021 formed a framework within the federal state that largely removed the basis for parliamentary functions. Essential for the analysis is the outline of the change in the multi-level governance that the pandemic brought about through the conference of the Chancellor and the 16 state prime ministers. The impact of this governmental crisis management politics are discussed for the most important state parliamentary functions, the oversight through co-governance, oversight by the opposition, legislation, and the public debate. The empirical basis is provided by media reports, non-public drafts, resolutions of the *Bund-Länder-Konferenz* and opinion polls. The article concludes with a recommendation to better prepare future emergency politics institutionally and to give state parliamentarism a place in this process.

## Einleitung

Deutschlands Performanz bei den Infektions- und Todeszahlen sowie den wirtschaftlichen und sozialen Folgen in Verbindung mit dem Coronavirus Disease 2019 (kurz: COVID-19) kann im EU27-Vergleich als zufriedenstellend bewertet werden (Ritchie et al. 2020; European Centre for Disease Prevention and Control 2021). Die Maßnahmen zur Eindämmung der Pandemie, die Bund und Ländern beschlossen haben, weisen ein gewisses Maß an Output-Legitimität (vgl. Scharpf 1999) auf, auch wenn sie seit Frühjahr 2021 aufgrund einer zunächst schleppenden Test- und Impfkampagne und der sich im Herbst desselben Jahres auftürmenden vierten Welle einige Kritik erfahren haben (vgl. Hassenkamp 2021; Pechtold 2021; Thurau 2021). Doch wie verhielt es sich mit der Legitimität durch demokratische Verfahren während der Pandemie (zur aktuellen Diskussion vgl. Decker 2020; Marschall 2020; Merkel 2020; Kersting 2021; Korte 2021; Florack et al. 2021)? Dieser Frage wird am Beispiel der Landesparlamente und deren Rolle bei der Entscheidungsfindung und Beschlussfassung innerhalb der föderalen Ordnung der Bundesrepublik Deutschland nachgegangen. Die These, mit der nach zwei Jahren Pandemie^1^ eine Zwischenbilanz gezogen werden soll, lautet, dass die Landesparlamente zentrale Funktionen (vgl. allgemein dazu Patzelt 2003; Marschall 2005; Dreischer 2006; Sebaldt 2009; Sieberer 2010) nicht in gewohnter Weise ausüben konnten, da sie in den langen „Stunden der Exekutive“, die während der Pandemiewellen schlugen, in ein institutionelles und prozedurales Hintertreffen gegenüber dem Notstandsregime der Bund-Länder-Konferenz (kurz: BLK) gerieten.

Die Analyse bezieht sich in theoretischer Hinsicht auf das ursprünglich für das Entscheiden in der Europäischen Union entworfene Emergency-Politics-Konzept von Jonathan White (2013). Zentral für den vorliegenden Aufsatz ist die Skizzierung der von der Pandemie evozierten Veränderungen des Regierens in der deutschen Mehrebenen-Demokratie in Gestalt der Bund-Länder-Konferenz. Anschließend werden in vier Einzelabschnitten Überlegungen und Argumente zu den potenziellen Auswirkungen der Regierungskrisenbewältigung auf zentrale Funktionen der Landesparlamente entwickelt. Beleuchtet werden die Parlamentsfunktionen, die potenziell am stärksten betroffen waren. Dies sind die Kontrolle durch Mitregieren seitens der Regierungsfraktionen, die Kontrolle der Regierung durch die Opposition, die Gesetzgebung sowie die Artikulationsfunktion bzw. das Herstellen von Öffentlichkeit. Empirische Grundlagen bilden Presseberichte, nichtöffentliche Dokumente der Bund-Länder-Konferenz^2^ sowie repräsentative Bevölkerungsbefragungen.

Die vorliegende Analyse versteht sich als ein Diskussionsbeitrag, der bei empirischen Untersuchungen einbezogen werden kann. Schließlich kann das schlichte Zählen von Parlamentsaktivitäten wie Parlamentsdebatten oder Kontrollinstrumenten wenig Aussagekraft beanspruchen, wenn der veränderte Entscheidungsrahmen eines Parlaments, dessen Kompetenzen durch vorgelagerte Gremien wie die BLK ausgeübt werden, nicht ausreichend wahrgenommen wird. Die Relevanz eines solchen Ansatzes lässt sich anhand eines Segelschiffs veranschaulichen, dem das Wasser abhandengekommen ist. Auf trockenem Boden kann die Besatzung einige Aufgaben weiter wahrnehmen und z. B. die Segel hissen, das Ruder drehen oder sich um die Verpflegung der Mannschaft kümmern. Womöglich können Einige diesem veränderten äußeren Zustand sogar für eine Weile etwas abgewinnen, weil manche Aufgabe nun leichter fällt. Aber all diese Aktivitäten sind letztlich nur eine Simulation ihrer ursprünglichen Funktionen. An das Ziel werden sie das Schiff nicht bringen.

Dass sich dieser Beitrag mit den Auswirkungen der deutschen Emergency Politics zur Corona-Pandemie auf die Landesparlamente und nicht den Deutschen Bundestag beschäftigt, hat zwei Gründe: Erstens finden die Landesparlamente in der Parlaments‑, Parteien- und Föderalismusforschung im Vergleich zum Bundestag offensichtlich weniger Beachtung, so auch in der aktuellen über die Auswirkungen der COVID-19-Pandemie auf den Parlamentarismus (vgl. bspw. Pyschny 2020; Siefken und Hünermund 2021).^3^ In international vergleichenden Bestandsaufnahmen stehen ebenfalls die nationalen Parlamente bzw. die Ersten Kammern im Vordergrund (vgl. bspw. Grabow 2021; Hildebrand 2020; IPU 2020a, b).^4^ Der aktuelle Forschungsbedarf für die Landesparlamente resultiert jedoch nicht nur aus einer akademischen Randlage (für Ausnahmen mit komparativer Ausrichtung vgl. Appeldorn und Fortunato 2021; Atzpodien 2020; Valentim und Widmann 2021), die für sich genommen eine wissenschaftliche Untersuchung nicht hinreichend rechtfertigen kann, sondern zweitens aus deren Bedeutung als regionale Hochburgen der föderalen Demokratie in Deutschland: Die Landesparlamente sind die einzigen direkt durch Wahlen legitimierten Institutionen der politischen Systeme der Länder (vgl. Mielke und Reutter 2012; Sturm 2020). Dementsprechend soll der Forschungsstand zu Parlamenten auf der Landesebene um den Krisenfall einer Pandemie erweitert und damit ein wissenschaftliches Puzzleteil für komparative Studien zum regionalen Parlamentarismus in Krisenzeiten erarbeitet werden.

Für den Ausblick werden Bevölkerungseinstellungen zum föderalen Entscheiden während der Pandemie herangezogen, denn eine Reform droht im Keim zu ersticken, wenn ihr der Rückhalt seitens der Bevölkerung versagt wird. Aufbauend auf der retrospektiven Analyse einer zeitlich begrenzten Ausnahmesituation soll damit auch zur Debatte über gangbare Ansätze zur demokratischen Beherrschung zukünftiger Emergency Politics beigetragen werden. Sollte tatsächlich ein „pandemisches Zeitalter“ (Habekuss 2021, S. 1) bevorstehen, das nach einem Leitartikel der Wochenzeitung DIE ZEIT mit der Verbreitung von COVID-19 erst begonnen habe, würden globale Herausforderungen, die beim Arten-, Umwelt- und Klimaschutz immer drängend werden und es wie bei der europäischen Finanz- und Wirtschaftskrise seit 2010 (vgl. LeDuc und Pammett 2013) oder der Geflüchtetenkrise 2014/15 (vgl. Mader und Schoen 2019) bereits waren, zur neuen Normalität parteienstaatlichen Regierens werden (vgl. White 2013; Florack 2021).

## Die deutsche Emergency Politics während der Pandemie-Wellen

Die Funktionsweise der Pandemie-Bewältigungspolitik in Deutschland kann mit dem theoretischen Ansatz der „Emergency Politics“ verstanden werden (vgl. White 2013; Kreuder-Sonnen und White 2021). Er basiert auf der Logik des Notstands, der die zeitgenössische Entscheidungsfindung in Demokratien wiederkehrend präge. Demnach stellen extreme Herausforderungen, die es zu managen bzw. zu lösen gelte, einen Freibrief für unkonventionelle Regierungsmaßnahmen dar. Die innenpolitische Emergency Politics – daneben wird eine unilaterale, multilaterale und supranationale unterschieden – zählt zur konventionellsten Form dieses „Exzeptionalismus“. Unter Berufung auf außergewöhnliche und drängende Handlungsnotwendigkeiten heben nationale Regierungen formelle oder informelle Beschränkungen auf, die sich aus demokratischen Kontrollmöglichkeiten, deliberativen Abwägungen, Verfahrensvorschriften und individuellen Rechten ergeben. Exekutive Ermessens- und Entscheidungsspielräume werden gegenüber der Verfassungsnormalität ausgeweitet.

Die Corona-Pandemie ließ diesen Exzeptionalismus in der gesamten Europäischen Union eskalieren (vgl. Kreuder-Sonnen und White 2021). Viele Regierungen haben in ihren Ländern die größten Einschränkungen von Freiheitsrechten seit dem Zweiten Weltkrieg vorgenommen. Dabei trug die Politik des Ausnahmezustands, selbst wenn sie aus binnenstaatlicher Perspektive ausschließlich national erscheinen mochte, auch supranationale Züge wie bei der gemeinsamen Beschaffung von Impfstoffen durch die Europäische Kommission. Die Reaktionen einzelner Staaten wie Grenzschließungen wirkten sich wiederum auf andere Staaten aus.

Die Bundesrepublik Deutschland, die aufgrund ihrer mehrfachen, sich auf unterschiedlichen Dimensionen überlagernden Gewaltenteilung nach dem Verständnis von Arend Lijphart (1999) als eine Konsensdemokratie eingeordnet werden kann, weist anders als Staaten wie Frankreich oder Großbritannien keine sehr starke Machtkonzentration auf der nationalen Ebene aus. Gouvernementaler Exzeptionalismus erforderte somit, dass die Bundesregierung und die Landesregierungen „an einem Strang“ zogen, um gemeinsam abgestimmt steuern zu können. Institutionell verlief dieses Zusammenspiel nach Annahme des Konzepts der Emergency Politics, das das „emergency regime“ als ein kollaboratives Phänomen einordnet.

In Form der außerkonstitutionellen Bund-Länder-Konferenz trat es vor allem während der sogenannten Lockdowns bzw. Shutdowns zum Vorschein, bei denen aufgrund hoher Infektionszahlen die politische Entscheidungsfindung auf der Bundesebene zentralisiert war. Währenddessen wurde das öffentliche, berufliche und private Leben in bisher nicht dagewesenem Umfang (bundesweite Betroffenheit), Intensität (für jedes Individuum spürbare Beschränkungen durch Kontaktverbote, Ausgangssperren oder Betriebsschließungen), Dramatik (in den Medien wurden an herausgehobener Stelle eine Zeitlang täglich neue Todeszahlen gemeldet) und Dauer (beispielsweise waren die Kindergärten und Schulen während der Lockdowns monatelang geschlossen) radikal heruntergefahren. Gerechtfertigt wurden die Schutzmaßnahmen damit, die Verbreitung des Virus durch Minimierung individueller Mobilität und Kontakte einzudämmen, um die Intensivbettenkapazität der Krankenhäuser nicht zu überlasten und letztlich Menschenleben zu schützen. Neben den vielen Politikfeldern, die in Reaktion auf die Pandemie betroffen waren, vor allem die Finanz‑, Sozial- und Wirtschaftspolitik, betrafen die Lockdowns daher besonders den staatlichen Umgang mit den im Grundgesetz garantierten Freiheitsrechten. Auf der politischen Ebene erfüllten die ungewohnte politische Entscheidungsunsicherheit, die drängenden Reaktionszwänge und der immense Erfolgsdruck geradezu beispielhaft die Bedingungen des Notstands-Exzeptionalismus.

Der vorliegende Beitrag bezieht sich auf die beiden bundesweiten Lockdowns im Untersuchungszeitraum von fast zwei Jahren beginnend im Februar 2020 und endend im November 2021. Der erste, kürzere Lockdown fand von März bis Mai 2020 statt. Der zweite, doppelt so lange Lockdown von November 2020 bis Juni 2021. Letzterer begann als „Lockdown Light“ und lief am Ende unter öffentlichen Druck als „Stotter-Shutdown“ mit beschränkten Öffnungen und Öffnungsrücknahmen im Frühsommer allmählich aus. Von den Lockdownphasen zu unterscheiden sind die jeweiligen Vor‑, Zwischen- und Nach-Lockdownphasen, in denen das Infektionsgeschehen abgeschwächt war und dieses von politischen Anpassungen der Corona-Regeln, auch landesspezifisch, begleitet wurde. Währenddessen glichen die binnenstaatlichen Entscheidungsprozesse wieder mehr der Prä-Corona-Zeit. Lag während des ersten bundesweiten Lockdowns eine beachtliche Regelungshomogenität zwischen den Ländern vor, war die Regelungslage und administrative Umsetzung während des zweiten heterogener (vgl. Behnke 2020).

## „Governance“ der gouvernementalen Spitzen von Bund und Ländern in einer Regierungsparteienallianz

**Figure 1:**
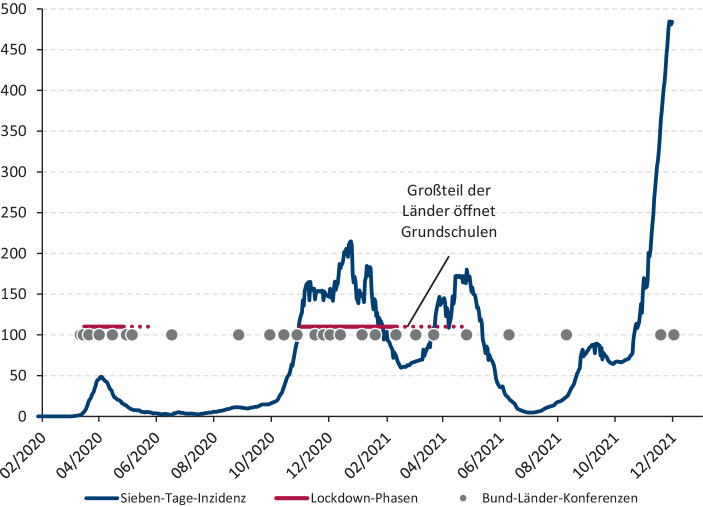

Für die politisch-administrative Pandemiebekämpfung in Deutschland hatte sich die Bund-Länder-Konferenz gebildet. Ihr gehörten die Bundeskanzlerin einerseits sowie die zwei Ministerpräsidentinnen und 14 Ministerpräsidenten bzw. Bürgermeister der Stadtstaaten (kurz: MP) andererseits an. Die BLK trat vor allem während oder unmittelbar vor und nach den Lockdowns in beinahe regelmäßigen Abständen zusammen. Während der ersten drei „Wellen“ verdichteten sich die Treffen (siehe Abb. 1). Sie fanden unter Ausschluss der Öffentlichkeit statt, zumeist als „Videoschaltkonferenz“ – so die Selbstbezeichnung – oder als Telefonkonferenz, seltener als persönliches Präsenztreffen. Am 2. Dezember 2021 tagte die BLK zum 26. Mal. Anfangs bezeichnete sie sich bloß als „Besprechung der Bundeskanzlerin mit den Regierungschefinnen und Regierungschefs der Länder“ (BLK-Beschlussprotokoll vom 12. März 2020). Mit rasch einsetzender Krisenroutine fand eine Selbstaufwertung zur „Konferenz“ (BLK-Beschlussprotokoll vom 1. April 2020) statt. Der damalige nordrhein-westfälische Ministerpräsident und Vorsitzende der Bundes-CDU Armin Laschet sprach im Zusammenhang mit der BLK von „unserer Methode der Governance“ (Henzler et al. 2021).

Nach dem Konzept der Emergency Politics bilden sich in schwerwiegenden Krisenfällen neue Akteurskonfigurationen heraus. Zwar knüpfte die Bund-Länder-Konferenz an bereits existierende horizontale Formate der exekutiven Politikkoordination an, insbesondere die „Ministerpräsidentenkonferenz“ (MPK), sie wurde aber strukturell erweitert, erstens um die vertikale Dimension der Bundeskanzlerin und zweitens um eine ebenenübergreifende Allianz aller in Deutschland auf der Bundes- oder Landesebene regierenden Parteien, sodass dieses Formation des Notstandsregimes als neuartig verstanden werden kann (zur Diskussion vgl. Waldhoff 2021).^5^

Erstens: In der Bund-Länder-Konferenz liefen die Fäden der in Deutschland – auch aus Gründen der Gewaltenteilung – föderal aufgeteilten gouvernementalen Macht zusammen: Hinter der Kanzlerin stand die Bundesregierung, hinter den „Landesfürsten“ die Landesregierungen. Die nach Art. 30 des Grundgesetzes funktionale Teilung von Staatsaufgaben wurde faktisch aufgehoben: Während im deutschen „Exekutivföderalismus“ die Gesetzgebung realiter hauptsächlich eine Bundesangelegenheit ist und für die Ausführung der meisten Bundesgesetze die Verwaltungen der Länder zuständig sind, bestand diese Machtaufteilung innerhalb der BLK nicht mehr. Dies veranlasste zu kreativen Begriffsschöpfungen wie „Intensivregierung“ (Bröchler 2021, S. 5), die jedoch insofern etwas in die Irre führen können, als mit der BLK zwar ein fraglos hochrangiges, aber immer nur koordinierendes Gremium der Eliten des Mehrebenen-Systems der Bundesrepublik geschaffen wurde. Auf die Ministerialbürokratien von Bund und Ländern konnte es nur mittelbar – seine Beschlüsse besaßen keine formale Bindekraft – und nicht als ein in sich geschlossener Akteur zugreifen, weder auf der Lenkungs- noch auf der administrativen Umsetzungsebene.

Als exekutives Spitzengremium des Bundes und der Länder umging die Bund-Länder-Konferenz die verfassungsrechtlich vorgesehenen Willensbildungs- und Entscheidungspfade innerhalb der föderalen Institutionenarchitektur, insbesondere das Bundesratsverfahren. Entsprechend der parlamentarischen Regierungslogik wusste die BLK die Parlamentsmehrheiten auf Bundes- und Landesebene hinter sich. Normalerweise bestehen zwischen den hierarchisch gegliederten Politikproduktionsarenen wechselseitige Abhängigkeits- und Abgrenzungsverhältnisse. Begrenztes politisches Steuerungspotenzial ist nur schwierig zu entfalten, gemeinsame Beschlüsse tragen zumeist Kompromisscharakter – Phänomene, für die der Begriff der „Politikverflechtungsfalle“ (Scharpf 1985) geprägt wurde. Vor diesem Hintergrund war während der Pandemie virulent, dass nicht ausreichend Zeit für das Verhindern oder Auflösen von Blockaden innerhalb des Verhandlungsregimes gegeben war. „Durchregieren“, wie es Angela Merkel als damalige Oppositionsführerin bei einer Bundestagsrede im Juli 2005 zur Auflösung des Bundestages in Aussicht gestellt hatte (vgl. Deutscher Bundestag 2005, S. 17.471), es während ihrer Kanzlerschaft aber immer nur mehr Wunsch als Wirklichkeit sein konnte (vgl. Zohlnhöfer 2010), wurde in den Pandemiejahren 2020 und 2021 das Gebot der Stunde. Mit dem kollektiven Akteur der Bund-Länder-Konferenz konnte in bisher unbekanntem Maße durchregiert werden.

**Figure 2:**
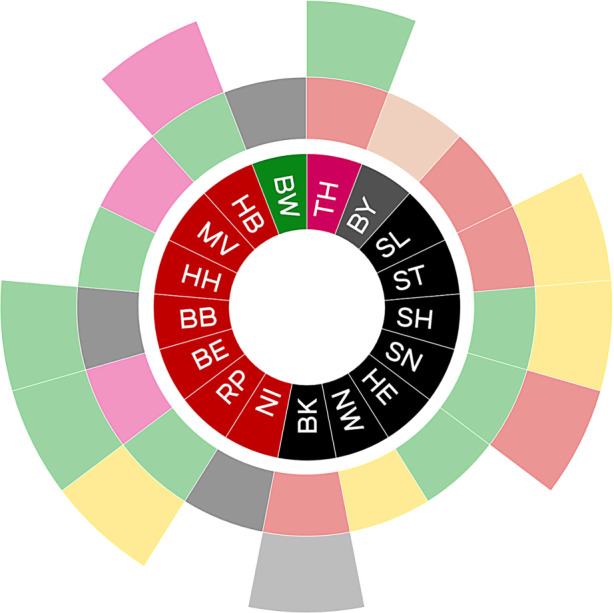

Zweitens: Das Spitzenpersonal aller regierenden Parteien in Bund und Ländern war in der Bund-Länder-Konferenz versammelt (siehe Abb. 2). Dieser Allparteienallianz gehörte mindestens eine Regierungspartei aus jedem der 16 Länder an, deren Bundesorganisation Teil der amtierenden Bundesregierung war.^6^ Außen vor blieb einzig die rechtspopulistische AfD, die nirgendwo Regierungsverantwortung trägt. Die Regierungsparteienallianz setzte ein weiteres Merkmal der föderalen Parteiendemokratie während der Lockdowns weitgehend außer Kraft, das als „Strukturbruchthese“ (Lehmbruch 2000) Eingang in die Forschung gefunden hat. Demnach gehen von den Parteien Vereinheitlichungsbestrebungen im Bundesstaat aus, die der föderalen Unterschiedlichkeit in bestimmten Politikfeldern zuwiderlaufen. Umgekehrt können Bundesparteipositionen durch Abweichungen von Landesregierungen im Bundesrat aufgebrochen werden (vgl. Müller et al. 2020; Souris und Müller 2020). Innerhalb der Bund-Länder-Konferenz trat dieses Spannungsverhältnis kaum zutage. Landes- und Parteiinteressen erschienen zumeist deckungsgleich. Ein Strukturbruch war kaum erkennbar.

Die Entscheidungsfindung in der Bund-Länder-Konferenz, die wie gezeigt neben den gouvernementalen „Vetospielern“ (Tsebelis 1995) auch alle parteipolitischen versammelt hatte, konnte nur im Konsens zustande kommen. Gelegentlich traten Schwierigkeiten bei der Beschlusssuche infolge divergierender Problemwahrnehmungen, auch angesichts regional unterschiedlicher Inzidenzwerte, und vereinzelter Profilierungsansinnen von Ministerpräsidenten, z. B. mit Blick auf eine bevorstehende Wahl im eigenen Land oder die lange Zeit offene Kanzlerkandidaten-Frage in der Union, auf. In dieses Bild passt, dass einzelne Ministerpräsidenten mitunter „auf die Bremse“ traten und schärfere Eindämmungsmaßnahmen verhinderten, selbst wenn sich die Kanzlerin dafür stark gemacht hatte. Mitunter gingen Länder auch über BLK-Beschlüsse hinaus und ordneten anstatt von Kontaktbeschränkungen eine Ausgangssperre an (vgl. Beucker 2021). Letztlich war aber der Einigungsdruck hoch und dadurch die Kompromissfindung erleichtert, denn die BLK versuchte auf eine Problemlage zu reagieren, für die manch historischer Superlativ^7^ bemüht wurde (vgl. Schwanholz 2021).

Bei den Verfahrensabläufen in der Bund-Länder-Konferenz, die anhand der nichtöffentlichen Beschlussvorlagen und Beschlüsse in ihren Grundzügen rekonstruiert wurden, lässt sich ein Austarieren von Einflusssphären feststellen. Im November 2020 hatten die Länder ein vorzeitiges Zusammentreffen der Konferenz, das von der Kanzlerin angesichts schnell ansteigender Infektionszahlen angestrebt worden war, abgelehnt und damit das „Vorpreschen“ Merkels demonstrativ ins Leere laufen lassen. Anstelle der anfangs üblichen „Beschlussvorschläge BUND“, die als Top-Down-Entscheidungsgrundlagen verstanden werden konnten, rückten Entwürfe der Länder, die seitens der Staatskanzleien unter Federführung des „MPK-Vorsitzlandes“ abgestimmt wurden (vgl. Lohse 2020). Aber auch bei dieser Bottom-Up-Entscheidungsvorbereitung sollte es nicht bleiben. Man fand schließlich zu einem Modus, der eine ausbalancierte Beteiligung der Länder und des Bundes vorsah. Anfang März 2021 war von der sogenannten „4er Runde“ ein Öffnungsplan aus dem Lockdown erarbeitet worden. Dieses Gremium setzte sich zusammen aus der Bundeskanzlerin, dem Vizekanzler Olaf Scholz, der schon zuvor an einigen BLK teilgenommen hatte (vgl. Henzler et al. 2021), dem Vorsitzenden der Ministerpräsidentenkonferenz Michael Müller und dem bayerischen Ministerpräsidenten Markus Söder. Somit waren nicht nur Spitzen der drei Parteien der Großen Koalition im Bund Teil des inneren Führungskreises der BLK, sondern er bildete auch eine gewisse Landesvarianz aus einem Flächenland im Süden und einem Stadtstaat im Osten ab.

## Die Funktionswahrnehmung der Landesparlamente im Zeichen der Emergency Politics der Bund-Länder-Konferenz

### Kontrollfunktion

Die parlamentarische Kontrolle der Exekutive stellt unter den Gesichtspunkten der in einer Demokratie unabdingbaren Gewaltenteilung und des Machtgefälles zugunsten der ressourcenstärkeren Regierung eine besonders wichtige Parlamentsfunktion dar (vgl. Lhotta 2009; Siefken 2018). Sie kann entweder im Vorfeld oder im Nachgang von Regierungsentscheidungen ausgeübt werden, hier herbeigeführt durch die Bund-Länder-Konferenzen. Für die Landesparlamente sticht die Kontrollfunktion im Vergleich zu den anderen Parlamentsfunktionen hervor (vgl. Höpcke 2014; Höhne 2019), auch da sie in den vergangenen Jahrzehnten einen Schwund an Gesetzgebungskompetenz zu verzeichnen hatten.^8^

Die Kontrollfunktion wird im Folgenden entlang ideengeschichtlicher Wurzeln im Republikanismus- oder Westminster-Modell zum einen in die Kontrolle durch Mitregieren und zum anderen in die Kontrolle der Opposition ausdifferenziert (vgl. Höreth 2021). Idealtypisch verläuft im parlamentarischen Regierungssystem analog zu dem ihm eigenen Dualismus aus Regierungs- und Oppositionsfraktionen eine Trennlinie bei der Wahrnehmung der Kontrollaufgaben (vgl. Hohendorf et al. 2020). Während für die Regierungsfraktionen eine eher „weiche“ Kontrolle durch Mitregieren typisch ist, ist es für die Oppositionsfraktionen eine eher „harte“ durch auf Abgrenzung setzendes Opponieren. Schematisch betrachtet befindet sich auf der einen Seite eine Kontrollvariante, die eher konstruktive Sachlösungen anstrebt und wissenschaftlich schwerer zu rekonstruieren ist, da sie vor allem in den Arkanbereichen von Politik stattfindet. Auf der anderen Seite befindet sich die Oppositionskontrolle, die mehr auf Öffentlichkeit abzielt. Vermeintliches Regierungsversagen soll vorgeführt und das eigene Profil als „Regierung im Wartestand“ geschärft werden (vgl. Schwarzmeier 2004; Schüttemeyer 2013).

#### Kontrolle durch Mitregieren

Die Kontrolle durch Mitregieren findet zwischen den Abgeordneten einer Regierungsfraktion und den Regierungsmitgliedern auf Grundlage einer geteilten Parteizugehörigkeit statt (für den Bundestag vgl. Schüttemeyer 1998). Aus Perspektive der Parteienforschung kann diese Akteurskonstellation zur „party in public office“ (Katz und Mair 2002) gezählt werden, die sich im Vergleich zu den anderen „party faces“ normalerweise durch bestimmte daseinsprägende Gemeinsamkeiten kennzeichnet. Verklammerte Parteidimensionen spielen für das Verständnis der eingeschränkten Kontrollmöglichkeiten von BLK-Beschlüssen eine wichtige Rolle: Zumeist sind die Ministerpräsident:innen Vorsitzende und Spitzenkandidierende ihrer Landespartei (siehe Tab. 1) und stützen sich auf die Regierungsfraktionen im Landesparlament.^9^ Während der Pandemie übten sich beide im Schulterschluss, der noch enger als in normalen Zeiten erschien; innerparteiliche Konflikte waren kaum auszumachen. In ihrer Bundespartei nehmen die Ministerpräsident:innen üblicherweise ebenfalls Führungsaufgaben wahr, die während der Pandemie infolge hoher TV-Präsenz medial aufgewertet wurden.^10^ Dann verklammern sie den parlamentarischen und gouvernementalen Strang des parteilichen Gesamtzusammenhangs in ihrem Bundesland mit dem des Bundes, der wiederum durch die BLK-Beschlüsse dominiert war. Summa summarum musste die Kontrolle durch Mitregieren in den Landtagen ganz im Zeichen der Bund-Länder-Konferenzen stehen.

**Table 1:** 

Land	Partei	MP	Funktion in Landespartei
Niedersachsen	SPD	Stephan Weil	Landesvorsitzender
Rheinland-Pfalz	SPD	Malu Dreyer	Beratendes Ex-Officio-Mitglied des Präsidiums des Landesvorstands
Berlin	SPD	Michael Müller	Von 2016 bis 2020 Landesvorsitzender
Brandenburg	SPD	Dietmar Woidke	Landesvorsitzender
Hamburg	SPD	Peter Tschentscher	–
Mecklenburg-Vorpommern	SPD	Manuela Schwesig	Landesvorsitzende
Bremen	SPD	Andreas Bovenschulte	– (Von 2010 bis 2014 Landesvorsitzender)
Baden-Württemberg	BÜNDNIS 90/DIE GRÜNEN	Winfried Kretschmann	Beratendes Ex-Officio-Mitglied des Landesvorstands, von 2002 bis 2011 Landesvorsitzender
Thüringen	DIE LINKE	Bodo Ramelow	–
Bayern	CSU	Markus Söder	Parteivorsitzender
Nordrhein-Westfalen	CDU	Armin Laschet	Landesvorsitzender
Hessen	CDU	Volker Bouffier	Landesvorsitzender
Sachsen	CDU	Michael Kretschmer	Landesvorsitzender
Schleswig-Holstein	CDU	Daniel Günther	Landesvorsitzender
Sachsen-Anhalt	CDU	Reiner Haseloff	Ex-Officio-Mitglied des Geschäftsführenden Landesvorstands
Saarland	CDU	Tobias Hans	Landesvorsitzender

Quelle: eigene Recherche (auf Homepages und in Satzungen der Landesparteien), Reihung der Länder wie in Abb. 2, Stand: 15. Oktober 2021

Das Konsensprinzip bei der Entscheidungsfindung in der Bund-Länder-Konferenz erforderte eine Flexibilität bei der Kompromisssuche. Wie wenig praktikabel eine förmliche parlamentarische Verpflichtung der Exekutive sowohl auf engere Positionen als auch breitere Zielvorgaben innerhalb definierter Korridore sein kann, wurde in der Europäisierungsforschung für den Bundestag und andere nationale Parlamente bei der Gesetzgebung in der Europäischen Union durch den „Ministerrat“ aufgezeigt (vgl. Sturm und Pehle 2012). Anders als bei internationalen Verhandlungssituationen erschien jedoch bei den binnenstaatlichen der BLK die Instrumentalisierung eines Parlamentsvorbehalts als Druckmittel, wie in Brüssel regelmäßig zu beobachten, innerhalb der Kultur des „kooperativen Föderalismus“ und noch mehr während der Corona-Ausnahmesituation fehl am Platze und wurde deshalb soweit erkennbar kaum praktiziert.

Bei der Exekutiv-Kontrolle durch die Regierungsfraktionen der Landtage im Anschluss an eine Bund-Länder-Konferenz bestand eine „Ratifikationssituation“ ähnlich wie im Allgemeinen bei der nachträglichen Kontrolle von bereits getroffenen Regierungsentscheidungen durch Parlamente (vgl. Schuett-Wetschky 2004). Abgesehen davon, dass die meisten Entscheidungen der BLK überhaupt keine Zustimmung seitens der Landesparlamente benötigten, dürfte sich praktisch auch kein Ansatzpunkt für die Ex-Post-Kontrolle ergeben haben. Ein Verhandlungspaket abzulehnen, das die „eigenen Leute“ mitgeschnürt haben, stünde nicht nur der Logik des parlamentarischen Regierens entgegen, sondern auch den eingespielten Verhandlungsmustern in der Mehrebenen-Demokratie. Welche Folgen es hat, wenn es dennoch geschieht, konnte Ende des Jahres 2020 in Sachsen-Anhalt bei einem erbitterten Parteienstreit über eine geplante Gebührenerhöhung für den öffentlich-rechtlichen Rundfunk beobachtet werden, der beinahe zum Ende der damaligen „Kenia-Koalition“ geführt hätte und seine Implikationen über das Land hinausreichten, weil womöglich die sich aus dem Bundesstaatsprinzip ergebende grundgesetzliche Rechtspflicht zu „bundesfreundlichem Verhalten“, die die wechselseitige Rücksichtnahme und das Zusammenwirken von Bund und Ländern vorsieht, berührt wurde.^11^

#### Kontrolle durch die Opposition

Die Opposition (in den Landtagen) unterlag während der Pandemie in besonderer Weise einem teils öffentlichen, teils selbstauferlegten Druck, „sich kooperativ zu verhalten“ (Höreth 2021, S. 127). Für den Bundestag wurde ein kooperatives und der Regierung zugewandtes Oppositionsverhalten während der ersten Pandemiewelle festgestellt (vgl. Louwerse et al. 2021). Konstruktive Zusammenarbeit, Gemeinwohlorientierung und staatspolitische Verantwortung wurden auch noch mehrebenen-parteiorganisatorisch befördert: Kritik einer oppositionellen Landtagsfraktion an der jeweiligen Landesregierung hätte mit Ausnahme der AfD immer auch die eigene Partei im Bund betroffen, zumindest deren Mitwirkung über die ihr zugehörigen Ministerpräsident:innen in der Bund-Länder-Konferenz. Zudem wurden inner- und intraparteiliche Differenzen durch Regierungskonsultationen der jeweiligen Oppositionsparteien zu den Maßnahmen der Pandemiebekämpfung eingehegt (vgl. Höreth 2021). Beispielsweise gab der damalige SPD-Oppositionsführer im Landtag von Schleswig-Holstein, Ralf Stegner, der dafür bekannt ist kein Blatt vor den Mund zu nehmen, circa ein Jahr nach Pandemiebeginn und vor dem Eindruck zunehmender Lockdown-Müdigkeit der Bevölkerung medial zu Protokoll: „Wir haben die Linie der Regierung bislang weitgehend mitgetragen, aber das ist keine Einbahnstraße“ (Müller 2021, S. 1).

**Figure 3:**
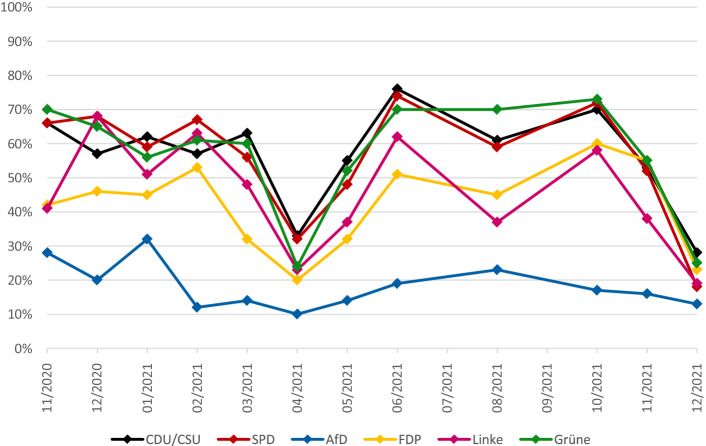

Die FDP, die der Bund-Länder-Konferenz über keinen Ministerpräsidenten angehörte, war nur mittelbar gegenüber deren Beschlüssen verpflichtet. Ihre Landesminister:innen verantworteten die von der BLK gesteuerte Corona-Landespolitik mit, beispielsweise als Teil des nordrhein-westfälischen Kabinetts. Wollten die Freidemokraten nicht an Glaubwürdigkeit einbüßen, worauf sie seit ihrem temporären Ausscheiden aus dem Bundestag im Jahr 2013 gesteigerten Wert legen (vgl. Höhne und Jun 2020), kamen sie gar nicht umhin, ihre im Parteiensystem angestammte Rolle als parteipolitische Anwältin von Freiheitsrechten während der Pandemie zu verteidigen. Zum einen war dies angesichts der geballten Medienkonzentration auf die BLK kein leichtes Unterfangen. Zum anderen erforderte ihr Status als Regierungspartei in drei bzw. vier Ländern und als Oppositionspartei im Bund eine geschickte Gratwanderung (vgl. Burger 2020). Bei Bevölkerungsbefragungen rezipierten Personen mit FDP-Neigung dieses Changieren tendenziell positiv und bescherten den Liberalen im Bundestagswahljahr 2021 demoskopischen und am Ende auch elektoralen Aufwind. Kontinuierlich überwogen bei ihnen die Anteile derjenigen, denen die „Corona-Maßnahmen“ von Bund und Ländern „zu weit“ gingen (siehe Abb. 3).^12^ Nur im AfD-nahen Spektrum waren ablehnende Haltungen noch häufiger vorzufinden.

Die AfD, als populistische Partei per definitionem in einer systemoppositionellen Stellung zu den anderen Parlamentsparteien (vgl. Jörke und Selk 2017), war sich zunächst uneins, ob sie die in der Bund-Länder-Konferenz konzertierten Regierungsmaßnahmen unterstützen oder sich gegen sie positionieren sollte (vgl. Fiedler 2020). Letzten Endes vermochte sie es nicht, ein Repräsentationsdefizit, das sich im Zuge einer restriktiven Notstandspolitik der Bund-Länder-Konferenzen aus Sicht eines Teils der Bevölkerung herausgebildet hatte, zu schließen. Stattdessen gründeten sich neue Parteien wie „dieBasis“, die bei der Bundestagswahl 2021 immerhin ein Zweitstimmenergebnis von 1,4 % erzielte und damit drittstärkste „Sonstige“ Partei wurde. Die wichtigste Opposition, allerdings eine außerparlamentarische und in sich sehr heterogene, wurden die Demonstrationen von selbsternannten „Querdenkern“. In deren Kreisen wurden neben esoterischen, weltverschwörerischen und religiösen Positionen auch solche von „Reichsbürgern“ und Rechtsextremen propagiert (vgl. Benz 2021; Frei und Nachtwey 2021; Grande et al. 2021; Pantenburg et al. 2021; Schließler et al. 2020).

### Gesetzgebungsfunktion

Die skizzierte Zusammensetzung und Macht des Verhandlungsregimes der Bund-Länder-Konferenz legen die Schlussfolgerung nahe, dass die in der Vergangenheit ohnehin schon eingeschränkte Gesetzgebungskompetenz der Landesparlamente während der Pandemie weiter geschwächt wurde. BLK-Beschlüsse konnten in den Ländern praktisch nur umgesetzt werden. Dies geschah vor allem über zeitlich befristetes Verordnungsrecht nach „Kettenverordnungsgebung“ (Pautsch und Haug 2020). Nicht beteiligt waren dabei die Landesparlamente, selbst bei Regelungsmaterien, die normalerweise in deren Zuständigkeit fallen (vgl. Behnke 2020). Sie befanden sich in einer ähnlich nachgeordneten Stellung wie das Bundesparlament. Zwar hat der Bundestag inzwischen schon viermal eine „epidemische Lage von nationaler Tragweite“ festgestellt. Dies geschah auf Basis des Infektionsschutzgesetzes (IfSG), an dem Bundestag und Bundesrat im Verlauf der Pandemie gemeinsam immer wieder Anpassungen vornahmen. Im Anschluss an diese High-Level-Entscheidungen mussten sich beide Kammern jedoch wieder mit einer peripheren Position außerhalb des Entscheidungszirkels der BLK zufriedengeben.

Das IfSG, mit dem der Bund von seiner konkurrierenden Gesetzgebungskompetenz nach Art. 74 Abs. 1 Nr. 19 GG Gebrauch gemacht hat, stellt die rechtliche Grundlage für das Verordnungsrecht während der COVID-19-Pandemie dar. Es räumt den Landesregierungen weitreichende Kompetenzen zu deren administrativen Bekämpfung ein, insbesondere durch die Ermächtigung zum Erlass von Rechtsverordnungen nach § 32 IfSG.^13^ In der Rechtswissenschaft wurde darüber diskutiert, wie die Beteiligung der Landesparlamente während der Pandemie hätte gestärkt werden können (vgl. Klafki 2020; Wissenschaftlicher Dienst des Schleswig-Holsteinischen Landtags 2020). Die Wahrnehmung der parlamentarischen Gesetzgebungskompetenz nach Art. 80 Abs. 4 GG wurde dafür als eine Möglichkeit gesehen. Im Falle einer Ermächtigung der Landesregierungen zum Erlass von Rechtsverordnungen durch ein Bundesgesetz, wie das IfSG, können Landesparlamente anstelle von Landesrechtsverordnungen Landesgesetze verabschieden. Obwohl die Regelungsqualität durch die Befassung eines Landesparlaments kaum abgenommen hätte und Legitimitätsgewinne wahrscheinlich gewesen wären, wurde dieser Weg kaum gewählt (vgl. Wissenschaftlicher Dienst des Landtags Rheinland-Pfalz 2020a, b; Jennewein und Korte-Bernhardt 2021). Dafür gab es mehrere Gründe, sowohl rechtswissenschaftliche als auch politikwissenschaftliche.

Erstens hätte der Pfad über Art. 80 Abs. 4 GG schlimmstenfalls bedeutet, dass eine im Bund beschlossene Regelungsmaterie mit eng gefasstem Rahmen nur im Nachhinein als Landesgesetz ausbuchstabiert worden wäre. Landesparlamente hätten dann nur mit geringen Freiheitsgraden selbst entscheiden können, auch nicht abweichend oder überhaupt nicht, sondern wären an die Norm der übergeordneten Ebene gebunden gewesen. Zweitens wurden in vielen Landtagen, wie z. B. in Baden-Württemberg, Parlamentsbeteiligungsinitiativen verhandelt und Gesetze verabschiedet, die den Parlamenten beim Erlass von Rechtsverordnungen ein Vetorecht, eine Zustimmungspflicht oder zumindest Informationsrechte einräumen (vgl. Amhaouach et al. 2021). Damit konnten Parlamente auch ohne den Art. 80 Abs. 4 GG an Entscheidungen über Maßnahmen teilhaben. Drittens hatten die Landesregierungen im Zuge der Änderungen des IfSG Mitte November 2020 selbst Pandemiebewältigungskompetenzen abgeben müssen, die damit auch den Landeslegislativen nicht mehr als Regelungsgegenstände zur Verfügung gestanden hätten. Die „Bundesnotbremse“ hatte Landkreise und kreisfreie Städte bei Überschreiten der Sieben-Tage-Inzidenz von 100 verpflichtet, dort eine Reihe konkreter und bundeseinheitlich festgelegter Schutzmaßnahmen durchzusetzen.

Politikwissenschaftlich gesehen wäre viertens für den deliberativ-legislativen Weg mehr Zeit als für den ministerialbürokratischen aufzuwenden gewesen. Zwar können parlamentarische Verfahren in Einzelfällen beschleunigt werden und es wurden innerparlamentarische Adaptionsvorschläge dazu unterbreitet, etwa durch einen „Sonderausschuss“ wie z. B. von der Thüringer FDP-Landtagsfraktion als Gesetzentwurf eingebracht (vgl. Thüringer Landtag 2020). Aber dies hätte das Problem knapper Zeit nicht strukturell gelöst, gerade bei einer Pandemiebekämpfung mit einer Vielzahl an schnell notwendigen Maßnahmen. Fünftens mangelte es den Parlamenten – noch mehr als den Ministerialapparaten – an Ressourcen und Expertise für tagesaktuelle Einschätzungen der pandemischen Lage und vor allem deren Bekämpfung.

### Öffentlichkeitsfunktion

„In akuter Not greifen parlamentarische Routinen nicht“, schreiben Claudia Landwehr und Armin Schäfer (2021, S. 136) und warnen vor schwindendem öffentlichen Rückhalt, wenn „technokratisch durchregiert“ wird. Dieser Gefahr konnte auch die Öffentlichkeitsfunktion der Landesparlamente, die maßgeblich zum Diskurs zwischen den Repräsentierenden (und ihren unterschiedlichen Parteipositionen) sowie den Repräsentierten (und ihren unterschiedlichen Politikansprüchen) eines Landes beiträgt (vgl. Höhne 2019), nur begrenzt begegnen. Unbestreitbar wurde seit Beginn der Pandemie eine Vielzahl an Parlamentsdebatten und Aktuellen Debatten bzw. Aktuellen Stunden (vgl. allgemein dazu Hohl 2018) abgehalten, teils unter neuartigen Bedingungen wie digitalen Sitzungen und abgesenkten Anwesenheitserfordernissen (vgl. Höhne 2020; Kroeber 2020; Jennewein und Korte-Bernhardt 2021). Infolgedessen wurde eine „aktive Rolle“^14^ der Landesparlamente ausgemacht, die jedoch nicht zwangsläufig eine relevante Rolle während der Lockdowns darstellen musste.

Vielmehr ist bezüglich der Wahrnehmung der Öffentlichkeitsfunktion davon auszugehen, dass es ihr vor der Rahmenbedingung eines sich rasant entwickelnden Infektionsgeschehen an ausreichend gesicherten Informationen mangelte. Dies betraf auch die Entscheidungsfindung der Bund-Länder-Konferenz zur Pandemiebewältigung, die wiederum hinter verschlossenen Türen stattfand und damit wenig kommunikative „Angriffsfläche“ bot. Zwar kann sicherlich keine Rede davon sein, dass die Landesregierungen ihren Informationspflichten gegenüber den Landtagen generell nicht nachgekommen wären, insbesondere durch eine Vielzahl an abgehaltenen Regierungserklärungen. Jedoch sah sich der niedersächsische Staatsgerichtshof in einem Urteil im März 2021 verpflichtet, die Landesregierung in Hannover mahnend an die exekutive „Bringschuld“ bei der Informationsversorgung des Landtags zu erinnern (vgl. Grunert 2021).

Der Öffentlichkeitsfunktion mangelte es wie gezeigt an einer Basis aus parlamentarischen Kontrollmöglichkeiten, parteipolitischen Gegensätzen und legislativen Entscheidungskompetenzen. Zwar konnten die Landesparlamente die erheblichen Grundrechtseingriffe, insbesondere während der Lockdowns, debattieren. Dies änderte jedoch nichts daran, dass jene mit Bundes- und Landesdekreten auf der Grundlage des Infektionsschutzgesetzes vorgenommen wurden, das außerhalb des legislativen Zuständigkeitsbereichs der Landesparlamente verabschiedet worden war. Dabei wurden in Anbetracht der Prämisse des parteiübergreifenden Zusammenhalts in Krisenzeiten, die sich institutionell durch die Regierungsparteienallianz der Bund-Länder-Konferenz ausdrückte, konkurrierende parteipolitische Lösungsoptionen zumeist nicht einmal ansatzweise herausgebildet.

Erst mit abnehmender Bindewirkung der BLK-Beschlüsse, wie sie seit Februar 2021 im Zuge rückläufiger Inzidenzwerte zu beobachten war, verbesserten sich die Bedingungen für das Ausüben der Öffentlichkeitsfunktion der Landesparlamente bzw. ihrer Fraktionen wieder. Gegensätze zwischen den Parteien oder auch zwischen einer Bundespartei und ihren Landesorganisationen konnten mehr zum Vorschein treten. War es während des ersten Lockdowns im Frühjahr 2020 für eine Landespartei schwierig (auch aufgrund der noch gänzlich unbekannten, akuten Bedrohungssituation), sich gegenüber der eigenen Bundespartei abweichend zu positionieren, wurde dies in der sich langsam vollziehenden Auslaufphase des zweiten Lockdowns leichter möglich. Kritik konnte sich auf „Sonderwege“ einer Landesregierung beziehen. Föderalstaatliche Verteilungskämpfe wurden wieder nach bekanntem Muster parteipolitisch aufgeladen.^15^

## Ausblick: Landesparlamente und nationale Emergency Politics auf föderaler Grundlage

Auf regionale Herausforderungen regionalspezifisch angemessen reagieren zu können, stellt – neben der Gewaltenteilung und staatlichen Integration ethnisch heterogener Gesellschaften – eine funktionale ideengeschichtliche Begründung für einen föderalen Staatsaufbau dar (vgl. Schultze 1990; Detterbeck et al. 2010; Riker 2008; Große Hüttmann 2020). Dass im deutschen Föderalismus – eher außerhalb der Lockdowns – auf unterschiedlich hohe Infektionszahlen mit von Land zu Land angepassten Krisenbewältigungsmaßnahmen reagiert wurde, wurde politikwissenschaftlich positiv gewürdigt (vgl. Behnke 2020; Kropp 2020; Münch 2020).

Diesen Weg hin zu einer stärkeren landespolitischen und damit auch parlamentarischen Verantwortung^16^ ging die sich formierende „Ampel-Koalition“ aus SPD, Bündnisgrünen und FDP im Bund, als sie die epidemische Lage nationaler Tragweite Ende November 2021 auslaufen ließ. Offenbar stellte für diese drei Parteien der während der Corona-Pandemie praktizierte Entscheidungsmodus der exekutiven Governance der Bund-Länder-Konferenz keine Versuchung zu einer weiteren Verstetigung dar. Jedoch ist das Bild mit Blick auf die politisch Handelnden nicht eindeutig. So kann eine MPK, die im März 2021 stattfand, als Vorzeichen eines gewachsenen Selbstbewusstseins exekutiver Spitzenkoordination gelesen werden. Sie begrüßte in ihrer Mitte die Präsidentin der Europäischen Kommission als Rednerin und unterstrich damit den eigenen politischen Stellenwert (vgl. Senatskanzlei des Regierenden Bürgermeisters von Berlin 2021).

Ohne ein umfassendes Bild der medialen Berichterstattung zeichnen zu wollen, bildete diese sicherlich sowohl affirmative als auch kritische Auffassungen gegenüber dem Krisenmanagement der Bund-Länder-Konferenz ab, die sich im Zeitverlauf der Pandemie wandeln oder relativieren konnten. Bund-Land- oder Land-Land-Gegensätze schienen jedoch während der Pandemie in den Medien zumeist auf ein geringeres Verständnis zu stoßen (vgl. Eckhard und Lenz 2020; Lenz et al. 2021), was sich zeigte, wenn die Rede von „Kleinstaaterei“, „föderale[n] Eitelkeiten“ oder manchem „Sonderweg“ war, wie z. B. beim eingeschränkten Aufenthaltsrecht in Mecklenburg-Vorpommern während des ersten Lockdowns (vgl. Focus Online 2020; Biallas 2020). Der „Chefökonom“ der Tageszeitung Handelsblatt, Bert Rürup, stellte sogar „grundlegende Schwächen im Staatsaufbau“ (Rürup 2020) fest.

Demoskopisch verdeutlichten sich Schwankungen bei der wahrgenommenen Krisenbewältigungskompetenz von Bund und Ländern. Augenscheinlich standen sie mit der medialen Berichterstattung über die Infektionsdynamik in Verbindung. Eigenständige Wege der Länder und somit auch mehr der Landesparlamente erfuhren in einer sich zuspitzenden Krisensituation weniger Unterstützung. Während der schrittweisen Normalisierung nach dem ersten Lockdown Anfang Juni 2020 war eine einheitliche Vorgehensweise der Länder nur von 55 % „eher befürwortet“ worden (infratest dimap 2020a, S. 5). Dagegen stimmten der Aussage: „Ich wünsche mir, dass die Bundesländer bei der Bekämpfung der Corona-Pandemie häufiger einheitlich vorgehen“ (infratest dimap 2020b, S. 11) Ende September 2020 78 % der Befragten eher zu. In jener Zeit stiegen die Infektionszahlen wieder an (siehe Abb. 1) und der Umgang mit der Pandemie lag in erster Linie noch in den Händen der Länder.

Je bedrohlicher die Pandemie erschien und je kritischer die Medienberichterstattung wurde, desto mehr sank die Unterstützung für eine landespolitische Krisenbewältigung. Dieser auf eine einfache Formel gebrachte Zusammenhang schließt an die Politische Kulturforschung zum Föderalismus in Deutschland an. Der föderalen Ordnung mit ihren Landesparlamenten wird zwar grundsätzliche Unterstützung seitens der Bevölkerung bescheinigt (vgl. Petersen 2019), die in jüngster Zeit sogar noch gewachsen sei (vgl. Köcher 2021). Jedoch zeigt sich bei näherem Blick eine eher fragile öffentliche Verankerung. Darauf deutet die als „Föderalismus-Paradox“ (Sturm et al. 2010) bezeichnete Konstellation zwischen föderalen Strukturen und ihren Outputs hin. Wenn letztere von Land zu Land verschiedenartig ausfallen, stoßen erstere auf mehr Unverständnis oder können sogar abgelehnt werden.

Welche Einsicht lässt sich aus den skizzierten Sichtweisen von Medien und Bevölkerung für die Aufgabenwahrnehmung der Landesparlamente im Zeichen zukünftiger nationaler Emergency Politics auf föderaler Grundlage gewinnen? Zweifelsohne sind sie Teil der politisch-kulturellen Rahmenbedingungen, die es bei einer Reform zu berücksichtigen gilt. Fraglich ist, ob aus ihnen eine Problemanerkennung hervorgehen kann, die zukünftige Notstandspolitik besser demokratisch einsetzt und über Parlamente an die Gesellschaft rückbindet. Immerhin wird darüber bereits eine Debatte geführt, die Eingang in die politische Auseinandersetzung gefunden hat (vgl. RND/dpa 2021).

Anstelle der ad hoc zusammengesetzten Bund-Länder-Konferenz braucht es ein reguläres Krisengremium als Back-Up-Option. Wenn Krisensituationen tatsächlich die neue politische Normalität darstellen, wie in der Literatur diskutiert (vgl. White 2013; Florack 2021), dann kann in einem Notstandsregime nicht auf die Einbindung demokratischer Elemente verzichtet werden. Dessen Konfiguration sollte auch für die Landesparlamente Kontrollmöglichkeiten vorsehen. Diese können dazu beitragen, dass sich für die exekutiven Eliten der öffentliche Erklärungs- und Rechtfertigungsdruck erhöht. Auch wenn in einer Krise durch eine nationale Regierungsparteienallianz das besonders für die parlamentarische Demokratie essentielle Wechselspiel zwischen Oppositions- und Regierungsfraktionen ausgehebelt wird, muss im Ausnahmezustand weiter um politische Alternativen gerungen werden können. Dabei kann den Parteien in subnationalen Parlamenten eine tragende Rolle zukommen.

## Fazit

Während der Wellen der Corona-Pandemie fand eine historisch beispiellose Machtkonzentration der Regierenden von Bund und Ländern in der Bund-Länder-Konferenz statt, die von einer Regierungsparteienallianz getragen wurde. Theoretisiert nach dem Konzept der Emergency Politics (vgl. White 2013) hat die BLK zentrale Herausforderungen der föderalen Parteiendemokratie – die „Politikverflechtungsfalle“ bei den politischen Mehrebenenverhandlungen sowie den „Strukturbruch“ zwischen parteilicher und föderaler Entscheidungslogik – außer Kraft gesetzt. Den Preis dafür mussten neben dem Bundestag, der sich nach Einschätzung von Wolfgang Merkel (2020) selbst entmachtet habe, die Landesparlamente zahlen. Er war selbst für den wahrscheinlichen Fall zu entrichten, dass die bundesstaatlich und zwischen den Parteien abgestimmten Corona-Krisenlösungen im Interesse der Landesparlamente und ihrer Fraktionen – mit Ausnahme der AfD und einer kritisch-konstruktiven FDP – gestanden haben.

Wie argumentativ dargelegt konnten die Landesparlamente in den Lockdown-Phasen wenig Kontrolle ausüben, weder oppositions- noch regierungsseits, kaum ihrer Gesetzgebungsfunktion nachkommen und nur begrenzt zur öffentlichen Debatte beitragen. Wenn überhaupt, waren sie mit der Corona-Politik vor allem nachträglich auf der Diskurs- und so gut wie kaum auf der parlamentarischen Entscheidungsebene befasst. Parlamentarische accountability herzustellen (vgl. Strøm 2000), schien schier unmöglich.

Lassen sich aus den Befunden Schlussfolgerungen für eine krisenfestere Zukunft der Landesparlamente ziehen? Zum einen ist der Gefahr zu begegnen, dass aus dem Ausnahmezustand der Massivkoordination über die Bund-Länder-Konferenz eine Default-Option während eines zukünftigen Notstands wird. Stattdessen braucht es ein extra dafür legitimiertes, d. h. demokratisch eingesetztes, Krisenkoordinationsgremium, das auch parlamentarische Kontrollmöglichkeiten vorsieht. Zum anderen ist es für die Landesparlamente unabdingbar, auch unter exzeptionellen Bedingungen, vorhandene Kontrollinstrumente anzuwenden und die öffentliche Debatte mitzugestalten. Bei Reformansätzen, Funktionseinschränkungen der Landesparlamente während einer Krisensituation Einhalt zu gebieten, ist die föderale Differenzaversion in der Bevölkerung, aber auch von Medien, als erschwerende Rahmenbedingung zu berücksichtigen.

Ziel dieses Aufsatzes war eine Auseinandersetzung mit den potenziellen Effekten der Emergency Politics während der Corona-Pandemie auf die Landesparlamente in Deutschland. Im Anschluss an die hier diskutierten Einschätzungen und Befunde kann zur weiteren Erkundung des neuartigen Phänomens der Governance über die Bund-Länder-Konferenz im Hinblick sowohl auf Tiefe als auch Breite weiterer Forschungsbedarf ausgemacht werden. Tiefergehende Untersuchungen könnten das Ausmaß von parlamentarischen Funktionseinbußen empirisch beleuchten, methodisch quantitativ wie qualitativ, etwa bei der Nutzung diverser parlamentarischer Kontrollrechte. Eine Fragestellung für breite ländervergleichende Studien wäre, inwiefern auch in anderen subnationalen Parlamenten Funktionseinbußen hinzunehmen waren und wie darauf reagiert wurde. Erkenntnisfortschritt für beide Richtungen kann zukünftig idealerweise zu einer Demokratisierung exekutiver Krisenpolitik beitragen.

## Footnotes

1 Erste Hinweise auf den Ausbruch des Corona-Virus in der chinesischen Stadt Wuhan lassen sich bereits im Dezember 2019 finden, wobei sich wahrscheinlich bereits im Herbst Menschen infiziert haben. Im Zuge seiner weltweiten Verbreitung wurde es am 27. Januar 2020 auch in Deutschland nachgewiesen (vgl. Jeglinsk 2021).

2 Recherchiert wurden die „Beschlussentwürfe“ und Beschlüsse der BLK sowie dazugehörige Pressemitteilungen über die kommerzielle Datenbank „Observer Gesundheitspolitik“. Sie wird u. a. von Pharmaverbänden und -unternehmen genutzt. Für die wissenschaftliche Forschung wurde dankenswerterweise ein kostenfreier Zugang ermöglicht. Ausgewertete BLK-Dokumente waren vom 12. März 2020, 16. März 2020, 22. März 2020, 1. April 2020, 15. April 2020, 30. April 2020, 5. Mai 2020 (23.30 Uhr), 6. Mai 2020 (Beschluss), 17. Juni 2020, 27. August 2020, 29. September 2020, 12. Oktober 2020, 14. Oktober 2020, 27. Oktober 2020 (22.29 Uhr), 28. Oktober 2020, 22. November 2020, 24. November 2020, 25. November 2020 (Beschluss), 13. Dezember 2020 (7.46 Uhr), 13. Dezember 2020 (Beschluss), 5. Januar 2021 (13.50 Uhr), 5. Januar 2021 (Beschluss), 18. Januar 2021 (21.00 Uhr), 19. Januar 2021 (12.40 Uhr), 19. Januar 2021 (Beschluss), 10. Februar 2021 (10.15 Uhr), 10. Februar 2021 (Beschluss), 1. März 2021 (19.10 Uhr), 2. März 2021 (15.00 Uhr), 3. März 2021 (7.30 Uhr), 3. März 2021 (21.00 Uhr), 3. März 2021 (Beschluss).

3 Allerdings haben einige Landtage ihre Wissenschaftlichen Dienste (z. B. in Rheinland-Pfalz) oder Forschende (u. a. in Baden-Württemberg, Brandenburg, Mecklenburg-Vorpommern, Niedersachsen, Sachsen, Thüringen) mit der Erstellung von Gutachten beauftragt (vgl. Wissenschaftlicher Dienst des Landtags Rheinland-Pfalz 2020a, b; Wissenschaftlicher Dienst des Schleswig-Holsteinischen Landtags 2020; für den Bundestag und das Europäische Parlament vgl. Wissenschaftlicher Dienst des Deutschen Bundestags 2020a, b; Wissenschaftlicher Dienst des Europäischen Parlaments 2020).

4 Beispielsweise hat die Zeitschrift „The Theory and Practice of Legislation“ (8. Jg., H. 1–2, 3) dem Thema Parlamente und Corona schon mehrere Ausgaben gewidmet *(Legislatures in the Time of Covid-19; Global Legislative Responses to Coronavirus 2020)* und die „Zeitschrift für Parlamentsfragen“ (52. Jg., H. 4) behandelt es in einem Themenschwerpunkt.

5 Wahrscheinlich würden die Ministerpräsident:innen dieser These widersprechen und die BLK als erweiterte MPK – bzw. im Terminus von Fritz W. Scharpf (1989) als Kooperationsform auf der „dritten Ebene“ – verstehen, bei der die Kanzlerin nur als Gast fungierte. Zum Hintergrund: Die MPK ist ein herausgehobenes Beispiel für die außerkonstitutionellen, wenngleich teils hochgradig institutionalisierten Praktiken der horizontalen Politikkoordination in Deutschland. Sie bildet das länderseitig zentrale informelle Gremium der Selbstorganisation zur gegenseitigen politischen Abstimmung. Nach ihrem Selbstverständnis sieht sie sich für die „großen Linien“ der Länder zuständig. Ihr Entscheidungsmodus ist der Konsens. Besteht in der MPK kein Interessengleichklang, beginnt die Kompromisssuche. Wenn seitens eines Lands eine bestimmte Lösung favorisiert wird, die aber nicht durchzusetzen ist, kann ein im Konsens herbeigeführter Kompromiss am Ende immer noch besser sein als eine Nicht-Entscheidung. Es besteht zwar kein Zwang zur Einigung, wohl aber eine Neigung dazu, denn alle Beteiligten teilen die Erfahrung, im selben Boot zu sitzen und vielleicht schon beim nächsten Treffen auf die Kompromissbereitschaft der anderen angewiesen zu sein. Erst wenn Beschlüsse der MPK in eine formale Gesetzesinitiative der Länder für den Bund überführt werden sollen, kommt der Bundesrat in Spiel. Derartige Vorhaben beanspruchen in der Zweiten Parlamentskammer große politische Durchschlagskraft, was auch daran erkennbar ist, dass sie dort zumeist einstimmig verabschiedet werden. Die Trennung zwischen der informalen Koordination und der formalen Entscheidung versucht man nach außen aufrecht zu erhalten, was auch daran zu erkennen ist, dass eine „MPK-Vorgeschichte“ in den Bundesratsdrucksachen zumeist nicht kenntlich gemacht wird (vgl. Nagel 2011; Müller et al. 2020).

6 Die Parteienbündnisse in Mecklenburg-Vorpommern (bis 15. November 2021), Niedersachsen und dem Saarland standen als Große Koalitionen sogar in parteipolitischer Kongruenz zum Bundeskabinett (bis 7. Dezember 2021), wenngleich die CDU in Schwerin und Hannover – anders als in Berlin und Saarbrücken – die dortige Regierung nicht anführte und auch keine Rücksicht auf die bayerische Schwesterpartei zu nehmen brauchte. Die Bündnisgrünen und Linkspartei waren über ihre beiden Ministerpräsidenten prominent in der BLK vertreten. Indirekt beteiligt waren die FDP als Juniorpartner in den Regierungen aus Nordrhein-Westfalen, Rheinland-Pfalz, Sachsen-Anhalt (ab 16. September 2021) und Schleswig-Holstein sowie ferner die FREIEN WÄHLER Bayern e. V., die mit der CSU koalieren.

7 In einer Fernsehansprache zu Beginn der Corona-Pandemie hatte Bundeskanzlerin Angela Merkel diese als „größte Herausforderung seit dem Zweiten Weltkrieg“ (Der Spiegel 2020) bezeichnet.

8 Die Entwicklung des bundesdeutschen Föderalismus hat bewirkt, dass den Landesparlamenten Kompetenzen abhandenkamen, die sich bei den Landesregierungen über deren Mitwirkung im Bundesrat und in verschiedenen Koordinationsgremien wie der Kultusministerkonferenz (KMK) wiederfanden (vgl. Carstensen und Schüttemeyer 2015; Laufer und Münch 1998; Thaysen 2004; abweichend vgl. Reutter 2017). Auch die europäische Integration trug dazu bei, dass eigenständige Gestaltungsspielräume weiter schrumpften (vgl. Abels und Eppler 2016).

9 Für die Landesminister:innen der Junior-Regierungsparteien ließen sich analoge Aussagen treffen, nur, dass sie nicht direkt der Bund-Länder-Konferenz angehörten und von daher etwas größere politische Freiheitsgrade gegenüber deren Beschlüssen beanspruchen konnten.

10 Bei der CDU tragen besonders die Ministerpräsident:innen zu einer engen Verklammerung der Landesparteien mit ihrer Bundespartei bei (für inhaltliche Positionsverklammerungen vgl. Müller 2013). Der damalige MP Armin Laschet führte die Bundespartei, MP Volker Bouffier war im Analysezeitraum einer von drei stellvertretenden Vorsitzenden. Die MP Daniel Günther, Tobias Hans, Reiner Haseloff und Michael Kretschmer wurden auf dem Bundesparteitag im Januar 2021 in das Präsidium, d. h. den engeren Führungskreis, gewählt.

11 Gegen die Beschlusslage der eigenen Koalition von CDU, SPD und Bündnisgrünen hatte die Unions-Landtagsfraktion an der Ablehnung der Gebührenerhöhung festgehalten, obwohl 15 Landesparlamente bereits zugestimmt hatten oder deren Zustimmung unmittelbar bevorstand. Die Gebührenerhöhung war vorläufig politisch vom Tisch, die Regierungskoalition nur mit Mühe gerettet und am Ende entschied das Bundesverfassungsgericht (vgl. Rath 2021).

12 Die starken Einbrüche bei der Zufriedenheit im April 2021 könnten auf Erwartungen, die nach der zweiten Welle gehegt wurden, zurückgeführt werden (vgl. Nienhaus 2021). Lockerungsversprechen konnten mit dem Aufkommen der Variante B1.1.7. und der dritten Welle (siehe Abb. 1 weiter oben) zunächst nicht aufrechterhalten werden (vgl. Erdmann und Kattwinkel 2021). Hinzu kam eine erratische Corona-Politik (vgl. Pausch 2021). Erst mit weiteren Lockerungsschritten nahm die Zustimmung im Frühsommer wieder zu bevor sie nach der Bundestagwahl im September 2021 und mit dem sich auftuenden Machtvakuum parteiübergreifend zurückging.

13 Das IfSG trat am 1. Januar 2001 in Kraft. Eine umfangreiche Novellierung wurde am 27. März 2020 im Zeichen von COVID-19 beschlossen. Eine weitere wesentliche Änderung folgte am 18. November 2020, die bislang letzte am 24. November 2021.

14 Muno, Wolfgang (2020): Die Rolle der Landesparlamente bei Maßnahmen zur Bekämpfung der Corona-Pandemie. Stellungnahme vor dem Rechtsausschuss des Landtags Mecklenburg-Vorpommern. Ausschussdrucksache 7/363, 26. November 2020.

15 Ende 2020 wurde über die Lastenverteilung zwischen Bund und Ländern bei der Finanzierung der Pandemie-Maßnahmen sowie die gestiegene Neuverschuldung des Bundes diskutiert. Der Vorsitzende der CDU/CSU-Bundestagsfraktion Ralph Brinkhaus forderte ein stärkeres finanzielles Engagement der Länder (vgl. Burger et al. 2020). Schließlich war der Gleichklang von Bund und Ländern in der BLK nicht unwesentlich durch die massiven Mitteleinsätze der Bundesregierung unterstützt worden, die der damalige Bundesfinanzminister Olaf Scholz als „Bazooka“ (Carstens 2021, S. 3) medial verschlagwortet hatte.

16 Im Koalitionsvertrag der „Ampel-Koalition“ mit dem Titel „Mehr Fortschritt wagen. Bündnis für Freiheit, Gerechtigkeit und Nachhaltigkeit“ heißt es dazu auf Seite neun: „Demokratie lebt vom Vertrauen in alle staatlichen Institutionen und Verfassungsorgane. Wir werden daher das Parlament als Ort der Debatte und der Gesetzgebung stärken“.
